# Autocrine Exosomal Fibulin-1 as a Target of MiR-1269b Induces Epithelial–Mesenchymal Transition in Proximal Tubule in Diabetic Nephropathy

**DOI:** 10.3389/fcell.2021.789716

**Published:** 2021-12-17

**Authors:** Yi-Chun Tsai, Wei-Wen Hung, Wei-An Chang, Ping-Hsun Wu, Ling-Yu Wu, Su-Chu Lee, Mei-Chuan Kuo, Ya-Ling Hsu

**Affiliations:** ^1^ School of Medicine, College of Medicine, Kaohsiung Medical University, Kaohsiung, Taiwan; ^2^ Division of General Medicine, Kaohsiung Medical University Hospital, Kaohsiung Medical University, Kaohsiung, Taiwan; ^3^ Division of Nephrology, Kaohsiung Medical University Hospital, Kaohsiung Medical University, Kaohsiung, Taiwan; ^4^ Drug Development and Value Creation Research Center, Kaohsiung Medical University, Kaohsiung, Taiwan; ^5^ Center for Liquid Biopsy and Cohort Research, Kaohsiung Medical University, Kaohsiung, Taiwan; ^6^ Division of Endocrinology and Metabolism, Kaohsiung Medical University Hospital, Kaohsiung Medical University, Kaohsiung, Taiwan; ^7^ Division of Pulmonary and Critical Care Medicine, Kaohsiung Medical University Hospital, Kaohsiung Medical University, Kaohsiung, Taiwan; ^8^ College of Medicine, Graduate Institute of Clinical Medicine, Kaohsiung Medical University, Kaohsiung, Taiwan; ^9^ College of Medicine, Graduate Institute of Medicine, Kaohsiung Medical University, Kaohsiung, Taiwan

**Keywords:** FBLN1, miR-1269b, epithelial–mesenchymal transition, exosome, proximal tubule, diabetic nephropathy

## Abstract

**Background:** Diabetic nephropathy (DN) is an increasing threat to human health and is regarded to be the leading cause of end-stage renal disease worldwide. Exosomes deliver biomolecule massages and may play a key role in cell communication and the progression of DN.

**Methods:** A cross-disciplinary study, including *in vivo*, *in vitro*, and human studies, was conducted to explore the cross-talk within proximal tubular epithelial cells (PTECs) in DN. Exosomal protein from PTECs treated with high glucose (HG) was purified and examined using liquid chromatography–tandem mass spectrometry (LC-MS/MS). Next-generation sequencing (NGS) was utilized to analyze RNAs extracted from PTECs from a type 2 diabetic patient and a normal individual. HK-2 cells were used to assess exosomal protein and its modulation and biofunction in DN. Normal individuals and type 2 diabetic patients were enrolled, and nondiabetic db/m mice and diabetic db/db mice were used to validate the molecular mechanism of exosomes in DN.

**Results:** HG stimulated PTECs to increase Fibulin-1 (FBLN1) expression, and PTECs secreted FBLN1 through exosome delivery, thereby inducing epithelial–mesenchymal transition (EMT) in PTECs. Transcriptome analysis found that FBLN1 expression was modulated by miR-1269b, which was downregulated by HG in HK-2 cells. While transfection of miR-1269b reversed FBLN1-mediated EMT in PTECs, miR-1269b inhibitor modulated the phenotype of PTECs toward mesenchymal type under normal glucose (NG) condition. Most importantly, urinary FBLN1 and exosomal miR-1269b levels were correlated with the severity of kidney injury in type 2 diabetic patients.

**Conclusion:** This study demonstrated the communication within PTECs through exosome transmission in an autocrine pattern. MiR-1269b–FBLN1 epigenetic regulatory network could be a potential therapeutic strategy to prevent the progression of DN.

## Introduction

Diabetes mellitus (DM) threatens human health and is an important public issue (Shaw et al., 2010). DM currently affects more than 463 million people worldwide, and the number of patients with DM is estimated to rise to 578 million by 2030 and 700 million by 2045 (Shaw et al., 2010). Diabetic nephropathy (DN) has been the leading cause of end-stage renal disease (ESRD), accounting for 40%–50% of all ESRD patients worldwide (Alicic et al., 2017). Patients with DN had high risks for cardiovascular or all-cause morbidity and mortality (Gembillo et al., 2021). Detecting DN onset or progression early and establishing a treatment strategy is the principle to prevent health and economic impact of DN.

The pathophysiologic mechanisms of DN are multifactorial and complicated. The typical hallmark of DN is the excessive deposition of extracellular matrix (ECM) proteins and epithelial–mesenchymal transition (EMT) in the tubulointerstitium, further leading to kidney fibrosis (Loeffler and Wolf, 2015). Recently, extracellular vesicles (EVs) have been reported to participate in the pathogenesis of DN and to be potential biomarkers for DN (Lu et al., 2020). EVs, including exosomes and microvesicles, are small spherical packages released by various living cells into the extracellular environment. They are important vectors for intracellular communication (Zhang et al., 2019). Exosomes, as small plasma membrane-derived vesicles with a diameter of 30–200 nm, could carry a variety of biomolecules (miRNAs, mRNAs, and proteins and lipids) derived from the origin of cells and transmit them to recipient cells (Chopra et al., 2019). Some studies have reported that exosomes mediated the cross-talk among different glomerular cells (Wang et al., 2018; Wu et al., 2017; Wu, et al., 2016). Exosomes carried Transforming Growth Factor Beta 1 (TGF-β1) mRNA derived from glomerular endothelial cells (GECs) that activate mesangial cells to promote renal fibrosis in DN (Wu et al., 2016). GECs and mesangial cells can interact with podocytes through the increased secretion of EVs enriched with TGF-β1 mRNA (Wu et al., 2017; Wang et al., 2018). However, the pathophysiologic role of exosomes in cross-talk within proximal tubular epithelial cells (PTECs) is not clear. Therefore, the aim of this cross-disciplinarily study was to investigate the role of cell–cell communication through PTEC-derived exosomal transduction to PTECs on remodeling in the microenvironment of DN.

## Materials and Methods

### Cell Lines and Cell Culture

Human kidney-2 (HK-2) cells (*ATCC* ^*®*^CRL-2190) were cultured in keratinocyte serum-free medium (Catalog 17005-042) plus 2% fetal bovine serum (FBS). Cells were treated with normal glucose (NG; 5.5 mM) and high glucose (HG; 25 mM) for the indicated times. Renal PTECs (RPTECs) of a type 2 DM (T2DM) patient and a normal individual (Lonza Walkersville Inc., MD, USA) were cultured in Clonetics^™^ REGM^™^ BulletKit^™^ (CC-3190) as in our previous study (Tsai et al., 2018).

### Exosome Isolation and Internalization by Cells Using Fluorescence Microscopy

Exosomes derived from HK-2 cells treated with NG and HG for 48 h were isolated and identified as in our previous study (Tsai et al., 2020). In brief, cells were seeded at a density of 2 × 10^6^ cells/100 mm dish and cultured for 48 h under NG or HG conditions in a cell culture medium containing 1% exosome-free serum (Life Technologies, Carlsbad, CA, USA). Exosomes were labeled with diluent C solution (20 μl, Life Technologies) and PKH26 (0.5 μl, Sigma-Aldrich Corp., St. Louis, MO, USA) for 5 min at room temperature and then incubated with HK-2 cells in the NG condition for 3 h. HK-2 cells were stained with Calcein-AM (1 μM, Life Technologies) for 30 min. The uptake of exosomes was found by using an immunofluorescence microscope (Nikon Instruments, Tokyo, Japan), and pictures were taken using a Nikon inverted fluorescence microscope (Eclipse TE200 microscope).

### Liquid Chromatography–Tandem Mass Spectrometry Analysis and Database Searching for Exosomal Protein Identification

The proteins of exosomes derived from the conditional medium of cells were purified using ExoQuick-TC (System Biosciences, Palo Alto, CA, USA) following the manufacturer’s protocols. Equal amounts of exosomal proteins from HK-2 cells treated with NG and HG for 48 h were prepared for liquid chromatography–tandem mass spectrometry (LC-MS/MS) analysis. Briefly, 10 μg of exosomal proteins from each sample was taken for reduction and alkylation by 100 mM of dithiothreitol and 100 mM of iodoacetamide, respectively, followed by trypsin digestion at 37°C overnight and desalting. Protein digests measuring 1 μg by trypsin (100 ng/μl) from each sample were then injected for Bruker Impact HD LC–quadrupole time-of-flight MS (LC-QTOF MS) (Bruker Daltonics, Bremen, Germany) with a NanoAcquity™ LC system (Waters, Milford, MA, USA) system. The instrument was run in V-mode with a mass resolution of ∼10,000. The accumulated data were analyzed using ProteinLynx Global Server software (PLGS2.3) using peptide and fragment mass accuracies of 25 ppm and 0.1 Da, respectively. Uniform carbamido methyl C and variable N-terminal acetyl, M oxidation, N deamidation, and C propionamide were selected as permitted modifications, up to one missed cleavage allowed and a maximum protein molecular weight of 250 kDa. This search engine was applied to the full Uniprot 15.0 database, human species. A search of SwissProt 57.1, *Homo sapiens* (human; 20,401 sequences) was also carried out using Mascot (2.3) search using the pkl peak list files generated in PLGS. An ion score cutoff of 32 is specified as identical or highly homologous according to Mascot outputs. A score ≥40 is typical in many reports, and we similarly considered peptides above this cutoff as positive hits. Peak lists were submitted to MASCOT software (version 2.5) against a forward and reverse *H. sapiens* SwissProt database. A minimum of 2 peptides per protein at a peptide and protein threshold of 95% were required for high confidence identification.

### RNA-Sequencing and Bioinformatics Analysis

As in our previous study (Tsai et al., 2018), TRIzol® was utilized to extract total RNA from cells performed by Welgene Biotechnology Company (Welgene, Taipei, Taiwan). The differentially expressed miRNAs of primary PTECs between the T2DM patients and normal individuals were defined at >2-fold change and >10 reads per million. The differentially expressed mRNAs between primary PTECs between the T2DM patients and normal individuals were defined at >2-fold change and >0.3 fragments per kilobase of transcript per million.

The functions and interaction networks of exosomal proteins were assessed using the STRING database (https://string-db.org/). The targets of specific miRNAs were predicted using two bioinformatics websites, TargetScan (http://www.targetscan.org) and miRmap (https://mirmap.ezlab.org), which classify potential specific miRNA targets according to miRmap scores and percentiles of Context++ score, and indicate the repression strength of a miRNA target. The networks of the selected miRNA targets were analyzed using Ingenuity Pathway Analysis (IPA) software (Ingenuity Systems, CA, USA), which provides “core analysis” of genes/proteins. The IPA software also offers miRNA–mRNA filter analysis to match miRNAs and their potential targets of mRNAs according to the repression strength.

### RNA Isolation and Reverse Transcriptase Quantitative PCR

Total RNA from cells and exosomes in the urine was isolated using TRIzol and TRIzolLS Reagent (Thermo Fisher Scientific, San Jose, CA, USA), respectively (Tsai et al., 2020). MiRNAs were reverse-transcribed using Mir-X™ miRNA First Strand Synthesis Kit (Takara, Maebashi, Japan). SYBR Green was used to analyze quantitative miRNA on the QuantStudio 3Q-PCR system. Relative expression levels of the miRNA in cells and exosomes were normalized to internal control U6. Relative expressions were presented using the 2^−ΔΔCt^ method. All primers used are listed in Supplementary Table S1.

### Transient Transfection

MiR-1269b mimic (100 nM), miR-negative control of mimic (miR-NC, 100 nM), miR-1269b inhibitor (50 nM), and miR-negative control of inhibitor (anti-miR-NC, 50 nM) (GE Healthcare Dharmacon, Lafayette, CO, USA), Fibulin-1 (FBLN1) siRNA (20 nM), and NC siRNA (20 nM) were transfected into cells using Lipofectamine™ RNAiMAX transfection reagent (Thermo Fisher Scientific, Waltham, MA, USA) following the manufacturer’s protocols.

### Western Blotting Analysis

The total protein of HK-2 cells was extracted using radio-immunoprecipitation assay (RIPA) lysis buffer (EMD Millipore, Burlington, MA, USA). The denatured protein was separated by 9%–11% sodium dodecyl sulfate–polyacrylamide gel electrophoresis (SDS-PAGE) and then transferred onto a polyvinylidene fluoride (PVDF) membrane by following blocking and immunoblotting with specific primary and secondary antibodies. Antibodies against exosome markers including *CD9*, *CD63*, and *CD81* (1:1,000, Cat EXOAB-KIT-1-SBI) from System Biosciences (Palo Alto, CA, USA); N-cadherin (1:1,000, Cat 610921), vimentin (1:1,000, Cat 550513), E-cadherin (1:1,000, Cat 610182), and fibronectin (1:1,000, Cat 610078) from BD Biosciences (Franklin Lakes, NJ, USA); FBLN1 (1:1,000, Cat ab54652) from Abcam (Cambridge, UK); and GAPDH (1:3,000, Cat mab74) from EMD Millipore (MA, USA) were used. The signals of blots were captured using Proteinsimple + Fluorchem Q system (Alpha Innotech, San Leandro, CA, USA). Densitometry of the blots was calculated using ImageJ software (USA).

### 3′UTR Luciferase Reporter Assay

Human embryonic kidney (HEK) 293 cells (1 × 10^4^/well) were co-transfected with pGL3-FBLN1-2-3′UTR luciferase plasmid/pRL-TK Renilla (8:1) or pGL3-FBLN1-3′UTR mutated (MT) luciferase plasmid/pRL-TK Renilla (8:1) with miRNA mimics (control mimic or miR-1269b mimic) using DharmaFECT Duo Transfection Reagent (Thermo Fisher Scientific, MA, USA) for 48 h. The activities of firefly and Renilla luciferase were then quantified using the Dual-Glo® Luciferase Assay System (Promega, Madison, WI, USA).

### Experimental Animals and Immunohistochemistry Stain of Mouse Kidneys

We purchased 6-week-old, pathogen-free male db/m mice for the non-DM animal model and db/db mice for the T2DM animal model from the National Laboratory Animal Center in Taiwan. All animal experiments in this study were approved by Kaohsiung Medical University and Use Committee (No. 107107). Body weight and blood glucose levels were monitored every week, and 24-h urine of mouse and kidney was collected at the 12th week. The kidney tissue sections were fixed in 4% paraformaldehyde for immunohistochemistry (IHC) staining. FBLN1 antibody (1:100, PAS-103841, Thermo Fisher Scientific, MA, USA) was used in IHC.

### Human Study Participants

Fifty-nine T2DM patients with estimated glomerular filtration rate (eGFR) ≥30 ml/min/1.73 m^2^ and 49 healthy volunteers were invited to participate from September 2016 to May 2017. DM was defined as a medical history of DM or the use of antidiabetic agents. Demographic and medical data were obtained from medical records and interviews with study participants. Blood and urine samples were taken after 12-h fasting for biochemistry studies at enrollment and stored in a −80°C freezer. Serum creatinine (Cr) was measured using the compensated Jaffé method in a Roche/Integra 400 Analyzer (Roche Diagnostics, Mannheim, Germany). The eGFR was calculated using the equation of the 4-variable Modification of Diet in Renal Disease Study (Levey et al., 1999). Concentrations of serum urea nitrogen (UN) were examined using the enzymatic method (Roche Diagnostics, Mannheim, Germany). This study was approved by the Institutional Review Board of Kaohsiung Medical University Hospital (KMUHIRB-G(I)-20180032). All participants provided written informed consent in accordance with the Declaration of Helsinki.

### Measurement of Urinary Albumin–Creatinine Ratio, Kidney Injury Molecule-1, Neutrophil Gelatinase-Associated Lipocalin, and Fibulin-1 Levels in Mice and Human

The levels of urinary albumin were assessed, as a marker of glomerular injury, and using the immunoturbidimetric assay with Tina-quant Albumin Gen.2 (ALBT2, Roche, USA). The levels of FBLN1 and proximal tubular injury indicators including kidney injury molecule-1 (KIM-1) and neutrophil gelatinase-associated lipocalin (NGAL) in the urine of mice and humans were measured using an ELISA kit (Cat MKM100 and Cat MKCN20 from R&D Systems, Minneapolis, MN, USA; Cat CSB-EL008452MO and Cat CSB-EL008452HU from CUSABIO, Houston, TX, USA) and Magnetic Luminex® Assay (Cat LXSAHM from R&D Systems, MN, USA; Cat CSB-EL008452HU from CUSABIO, TX, USA), respectively. Concentrations of urine Cr were examined using the enzymatic method (Roche Diagnostics, Mannheim, Germany). Concentrations of urinary albumin, KIM-1, NGAL, and FBLN1 were corrected by urine Cr before statistical analysis.

### Statistical Analysis

Continuous variables were expressed as mean ± standard error of the mean (SEM) or median (25th, 75th percentile) as appropriate, and correlations among continuous variables were examined using Spearman’s correlation analysis. Categorical variables were expressed as percentages, and differences were tested using the chi-square test. The differences in continuous variables between groups were analyzed using Student’s *t*-test or one-way ANOVA, followed by the post-hoc test with a Tukey’s correction. Statistical analyses were conducted using SPSS version 18.0 for Windows (SPSS Inc., Chicago, IL, USA) and GraphPad Prism 9.2.0 (GraphPad Software Inc., San Diego, CA, USA). Statistical significance was set at a two-sided *p*-value of <0.05.

## Results

### Identification of Differentially Exosomal Proteins Derived From Proximal Tubular Epithelial Cells in Diabetic Nephropathy

**FIGURE 1:**
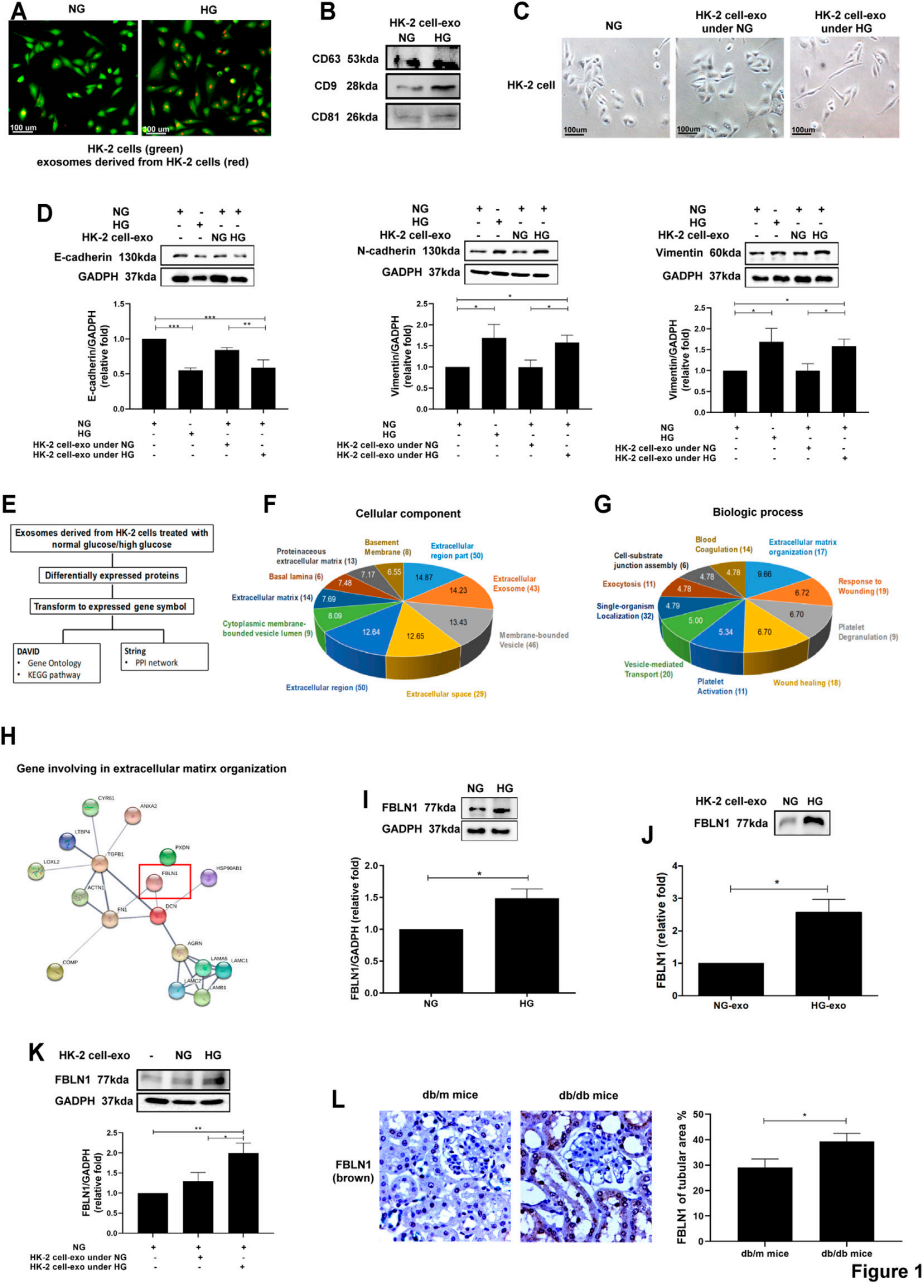
Identification of differentially exosomal proteins derived from proximal tubular epithelial cells (PTECs) in diabetic nephropathy (DN). **(A)** Detection of normal glucose (NG; 5.5 mM)- or high glucose (HG; 25 mM)-treated HK-2 cell-derived exosomes uptake by HK-2 cells using immunofluorescence stain. **(B)** Examination of the markers of exosomes derived from HK-2 cells treated with NG or HG for 48 h using Western blotting. **(C)** The morphology of HK-2 cells treated with NG or exosomes derived from HK-2 cells under NG or HG for 48 h (exosomes: HK-2 cell = 5:1) was examined using a light microscope. **(D)** Epithelial–mesenchymal transition (EMT) markers were assessed in HK-2 cells treated with NG, HG, or exosomes derived from HK-2 cells under NG and HG conditions for 48 h using Western blotting. **(E)** Flowchart of identification of potential exosomal proteins derived from HK-2 cells treated with NG and HG for 48 h using liquid chromatography–tandem mass spectrometry (LC-MS/MS) and following bioinformatics analysis. **(F,G)** Gene ontology of cellular component and biological process of exosomal proteins derived from NG- and HG-treated HK-2 cells. The pie chart indicates the-Log10 (false discovery rate (FDR)) of each term, and the numbers that are shown at the outside of each pie segment indicate the number of genes involved in each term. **(H)** The protein–protein interaction network analysis of genes associated with extracellular matrix organization. STRING database (version 10) was used in the bioinformatics analysis. **(I,J)** Fibulin-1 (FBLN1) expression in HK-2 cells treated with NG and HG for 48 h, and with exosomes derived from HK-2 cells under NG and HG conditions for 48 h. **(K)** The FBLN1 protein level in HK-2 cells treated with NG or exosomes derived from HK-2 cells under NG and HG conditions for 48 h (exosomes: HK-2 cells = 5:1). **(L)** The expression of FBLN1 in the proximal tubules of kidneys in mice is shown. The kidney sections of nondiabetic db/m mice (*n* = 3) and diabetic db/db mice (*n* = 3) were stained with FBLN1 (brown). The bar graph represents the mean ± SEM of at least three independent experiments. ^*^ *p* < 0.05, ^***^ *p* < 0.001 by Student’s *t*-test.

To investigate the autocrine delivery of exosomes, exosomes were collected from HK-2 cells treated NG and HG for 48 h, and the characteristics of exosomes were clarified as in our previous study (Tsai et al., 2020). Immunofluorescence revealed that HK-2 cells took up exosomes derived from HK-2 cells under NG or HG conditions (Figure 1A). Exosome markers, including CD63, CD9, and CD81, were expressed in NG- or HG-treated HK-2 cell-secreted exosomes (Figure 1B). In addition, the morphology of HK-2 cells treated with HG-treated HK-2 cell-derived exosomes changed from the cuboidal epithelial structure to the elongated mesenchymal shape (Figure 1C). EMT was induced in HK-2 cells treated with HG-treated HK-2 cell-derived exosomes, as well as treated with HG (Figure 1D).

To further investigate the pathophysiologic role of exosomal proteins derived from PTECs in DN, exosomes were collected from HK-2 cells treated NG and HG for 48 h. Protein samples from HK-2 cell-derived exosomes were examined using LC-MS and then were analyzed by bioinformatics (Figure 1E). One hundred thirteen proteins were identified in exosomes derived from HG-treated HK-2 cells. STRING database (version 10.0, https://string-db.org/) revealed that gene ontology of cellular component and biological process of exosomal proteins derived from HG- or NG-treated HK-2 cells revealed that these differentially expressed genes were associated with the extracellular region and ECM organization (Figures 1F,G). The STRING database was utilized to identify the potential interaction among 17 differentially expressed exosomal proteins related to ECM organization (Table 1) and found that FBLN1 might have an interaction with fibronectin-1 (FN1), as a principal marker of ECM, in ECM organization (Figure 1H). In addition, we have analyzed different mRNAs of PTECs of the patients with T2DM compared with those of the normal individuals in our previous study (Tsai et al., 2018), and we also found that FBLN1 mRNA expression was elevated in PTECs of the patients with T2DM (fold change of 7.14 as compared with the normal individuals). Others did not reveal significantly different expressions between the two groups. Thus, we further examined the pathophysiologic role of FBLN1 in DN. FBLN1 protein expression was increased in both cell lysate (Figure 1I) and exosomes extracted from HG-treated HK-2 cells (Figure 1J). HG-treated HK-2 cell-derived exosomes enhanced FBLN1 protein levels in HK-2 cells (Figure 1K). Furthermore, the levels of expression of FBLN1 at protein expression level were higher in the proximal tubules of kidney sections of diabetic db/db mice than those of nondiabetic db/m mice (Figure 1L). These findings supposed that HG increased FBLN1 expression and secretion by exosomes in PTECs.

**TABLE 1:** Biological process of exosomal proteins derived from HK-2 cells measured by LC-MS/MS according to STRING database.

Pathway description	Observed gene count	Matching proteins
Extracellular matrix organization	17	ACTN1, AGRN, ANXA2, COMP, CYR61, DCN, **FBLN1**, FN1, HSP90AB1, LAMA5, LAMB1, LAMC1, LAMC2, LOXL2, LTBP4, PXDN, and TGFB1

Note. LC-MS/MS, liquid chromatography–tandem mass spectrometry.

### Fibulin-1 Induced Epithelial–Mesenchymal Transition in Proximal Tubular Epithelial Cells

**FIGURE 2:**
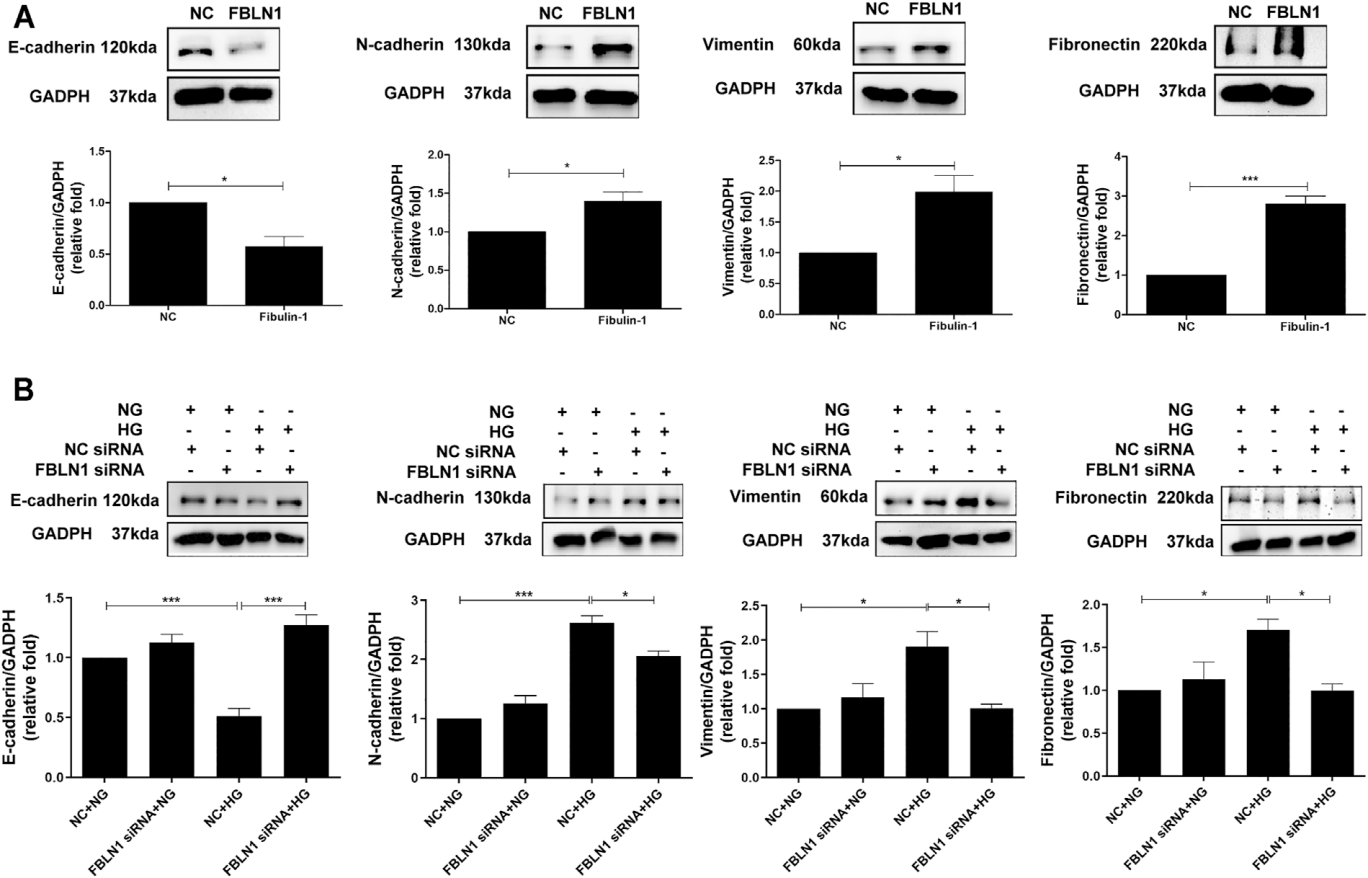
Fibulin-1 (FBLN1) induced epithelial–mesenchymal transition (EMT) in proximal tubular epithelial cells (PTECs). **(A)** EMT markers, including E-cadherin, N-cadherin, and vimentin, and extracellular matrix marker (ECM; fibronectin) levels were assessed in HK-2 cells treated with normal control (NC) and FBLN1 protein (300 ng/ml). **(B)** Cells were transfected with FBLN1 siRNA (20 nM) or control siRNA for 24 h. After transfection, the cells were treated with normal glucose (NG) or high glucose (HG) for 48 h to examine EMT and fibronectin expression using Western blotting. *p < 0.05, **p < 0.01, and ***p < 0.001 by Student’s *t*-test or ANOVA followed by the post-hoc test adjusted with Tukey’s correction.

EMT is one of the major pathophysiologic mechanisms of proximal tubular injury in DN (Loeffler and Wolf, 2015). We investigated whether FBLN1 contributes to EMT in HK-2 cells. A decreased expression of E-cadherin and elevated expression of N-cadherin, vimentin, and fibronectin were found in HK-2 cells treated with FBLN1 protein (Supplementary Figure S1A; Figure 2A). Furthermore, silencing of FBLN1 by siRNA transfection was performed in HK-2 cells (Supplementary Figure S1B). Suppression of FBLN1 not only reversed the decreased expression of E-cadherin and increased expression of N-cadherin and vimentin in HK-2 cells treated with HG (Figure 2B) but also reduced fibronectin elevation induced by HG. These results suggested that FBLN1 promoted EMT caused by HG in HK-2 cells.

### Fibulin-1 as a Direct Target of MiR-1269b in Proximal Tubular Epithelial Cells of Diabetic Nephropathy

**FIGURE 3:**
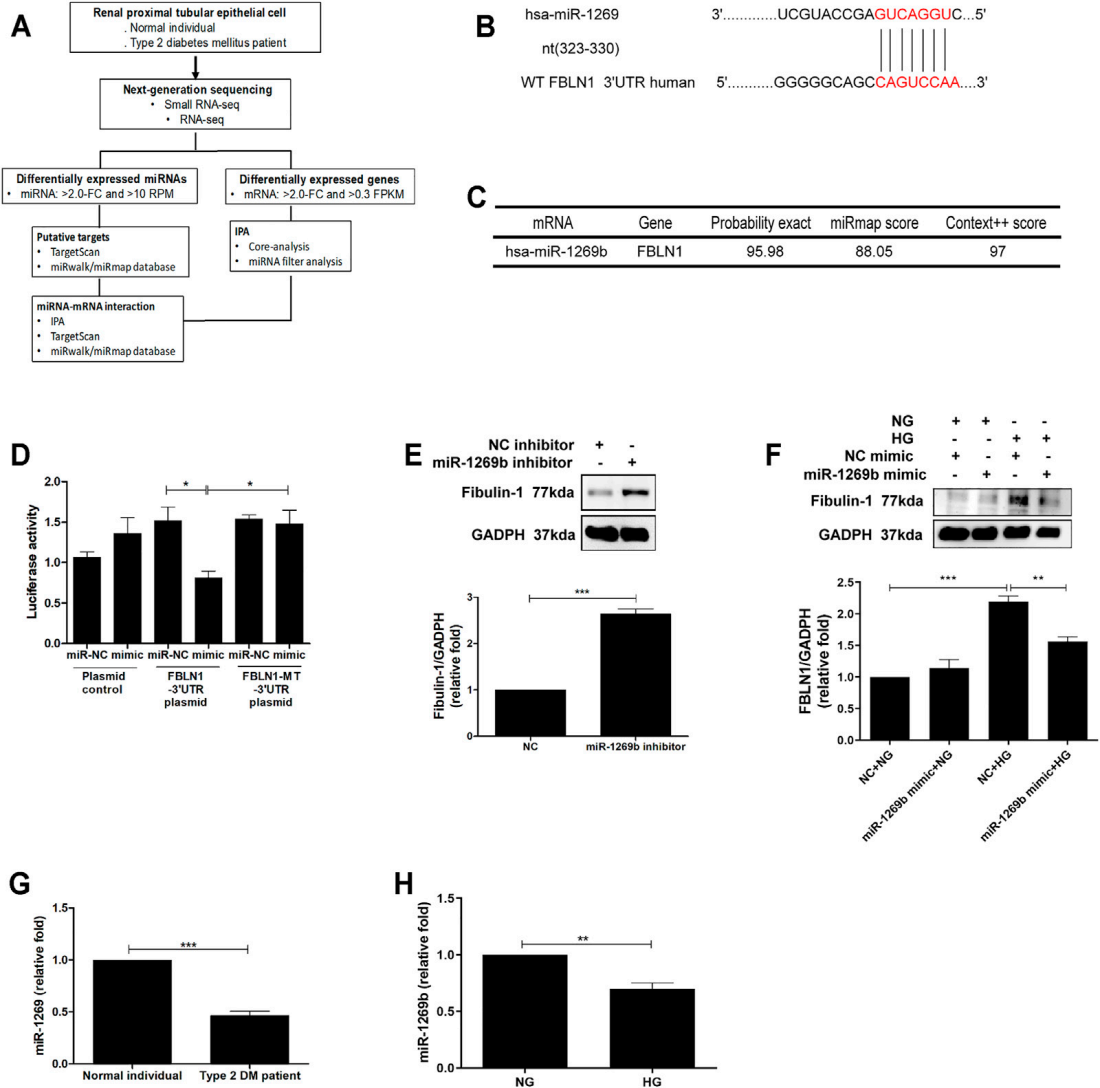
Fibulin-1 (FBLN1) as a direct target of miR-1269b in proximal tubular epithelial cells (PTECs) of diabetic nephropathy (DN). **(A)** Flowchart of identification of potential miRNAs from renal PTECs obtained from a normal individual and a type 2 diabetic patient by next-generation sequencing (NGS) and following bioinformatics analysis. (**B)** A schematic representation of sequence alignment of FBLN1 mRNA 3′UTR based on TargetScan (version 7.1). **(C)** The predictive binding score of miR-1269b on 3′UTR of FBLN1 mRNA according to miRmap and TargetScan (version 7.1) database. **(D)** The activity of FBLN1 3′UTR luciferase reporter plasmid was repressed by exogenous miR-1269b. HEK 293 cells were co-transfected with pGL3-FBLN1-3′UTR luciferase plasmid/pRL-TK Renilla (8:1) or pGL3-FBLN1-3′UTR MT luciferase plasmid/pRL-TK Renilla (8:1) with various miRNA mimics (control mimic or miR-1269b mimic) using DharmaFECT Duo Transfection Reagent. After 48 h, both firefly and Renilla luciferase activities were quantified using the Dual-Glo® Luciferase Assay System. **(E,F)** After transfection with miR-1269b inhibitor (50 nM) and miR-1269b mimic (100 nM) for 24 h, HK-2 cells were treated with normal glucose (NG) or high glucose (HG) for 48 h, and then FBLN1 expression was measured using Western blotting. **(G,H)** MiR-1269b expression in renal PTECs of a normal individual and a type 2 diabetic patient and HK-2 cells treated with NG and HG for 48 h. MiR-1269b levels were assessed by quantitative real-time PCR. The bar graph represents the mean ± SEM of at least three independent experiments. RPM, reads per million. *p < 0.05, **p < 0.01, and ***p < 0.001 by Student’s *t*-test or ANOVA followed by the post-hoc test adjusted with Tukey’s correction. NT, wild type; MT, mutation.

Based on our findings that FBLN1 induced EMT in PTECs of DN, we investigated whether the increase in FBLN1 is via an epigenetic mechanism in the process of PTEC EMT. As in our previous study (Tsai et al., 2018), the RNA profiles of RPTECs of a normal individual and a patient with T2DM were established using RNA-sequencing and analyzed using *in silico* websites (Figure 3A). Different miRNAs (three upregulated miRNAs and 64 downregulated miRNAs) and mRNAs (280 upregulated mRNAs and 322 downregulated mRNAs) with a significant 2-fold change were found in RPTECs of the patients with T2DM compared with those of the normal individual. We utilized miRNA target filter of IPA to search miRNA regulators of FBLN1 and found that miR-1269b targeted FBLN1 and regulated FBLN1 expression (Table 2). Core analysis of IPA also revealed that the predicted target gene of miR-1269b participated in the pathologic network of cellular growth and proliferation, and FBLN1 was one of the genes involved in this pathway (Table 3). Next, we used TargetScan (7.1 version) to predict the biological targets of miRNAs, which revealed that miR-1269b targeted positions 323–330 of FBLN1 3′UTR (Figure 3B), with the context score percentile of 97 (Figure 3C), which meant that the miR-1269b–FBLN1 link was highly practicable. Luciferase receptor assay showed that the miR-1269b is directly bound to 3′UTR of FBLN1 (Figure 3D). Transfection of miR-1269b inhibitor increased the expression of FBLN1 protein in HK-2 cells under the NG condition (Figure 3E), and elevated FBLN1 protein induced by HG was suppressed in HK-2 cells after transfection of miR-1269b mimic (Figure 3F). MiR-1269b level was decreased in RPTECs of a T2DM patient as compared with that of a normal individual (Figure 3G). In addition, HG reduced miR-1269b expression in HK-2 cells (Figure 3H). In short, HG reduced miR-1269b expression and resulted in FBLN1 upregulation in PTECs.

**TABLE 2:** Potential microRNA–mRNA interactions identified in diabetic PTECs.

miRNA	Precursor	Log2 ratio	Fold change	DM seq (norm)	Non-DM seq (norm)	DM read count	Non-DM read count	Target gene	Fold change
hsa-miR-1269b	hsa-mir-1269b	−1.58	−3.00	3.98	11.95	46	133	FBLN1	2.836

Note. PTECs, proximal tubular epithelial cells; DM, diabetes mellitus.

**TABLE 3:** The network analysis of predicted target genes of miR-1269b according to IPA.

Top diseases and functions	Score	Focus molecules	Molecules in network
Cell cycle, cellular growth and proliferation, organismal injury, and abnormalities	39	46	ALDH1A3, ALDH2, ALDH3A1, ALKBH1, APOC2, AREG, ARL2, ARMC10, BCL6, BMI1, C1orf115, C9, CAPN2, CCDC47, CCL11, CDKN1A, CDKN2AIP, CFL2, CORO6, COTL1, CTBP1DT, CYP2J2, DLEU1, DNAJC6, DUSP13, DUT, DYNLT3, E2F1, E2F6, E4F1, EYA2, FAM167A, FAM198B, **FBLN1**, FBXL2, FBXO11, FBXO46, FN1, FOXG1, FUT1, GCHFR, GLIPR1, GPR87, GSTT1, HENMT1, HIST2H4B, HLAJ, HS3ST1, IL7, JPH3, JUN, KAT6A, KAT8, KLF17, KRT20, LAG3, LINC00475, LIX1, LSS, LUM, MAFF, MAGEB2, MAGEC2, MBNL2, MED13L, MED22, MEG3, miR-142, miR-142-3p, miR-22-3p, miR-30c-5p, MSI1, MT-CO2, MT1L, MYO9B, NEUROG1, NID1, NINJ1, NIPAL4, NOC2L, NPFFR2, NTRK2, PALMD, PCDHGA11, PDZD2, PHC1, PLAGL2, POLR2A, PRKCA, PRKCD, PRSS23, PSMA3, PSMB8, PTAR1, PTPN1, PYHIN1, RAD23A, RASSF3, RASSF6, RBBP5, RGS14, RHOBTB3, RIPOR2, RNF126, SATB1, SCO2, SELENOP, SEMA3C, SERPINB8, SLC38A2, SLC43A3, SMARCA4, SNAPC3, SOGA1, SQLE, SRY, STAM2, STAT5A, TAGLN2, TBP, TEP1, TMEM117, TNF, TNFRSF10C, TP53, TP53RK, TPM4, TRIM28, TRIM39, TRIM41, TSPAN13, TUBA3C/TUBA3D, ULK2, USP42, VKORC1L1, VRK2, VTA1, WARS, WT1, YEATS4

Note. IPA, Ingenuity Pathway Analysis.

### MiR-1269b Reduced Epithelial–Mesenchymal Transition in Proximal Tubular Epithelial Cells of Diabetic Nephropathy

**FIGURE 4:**
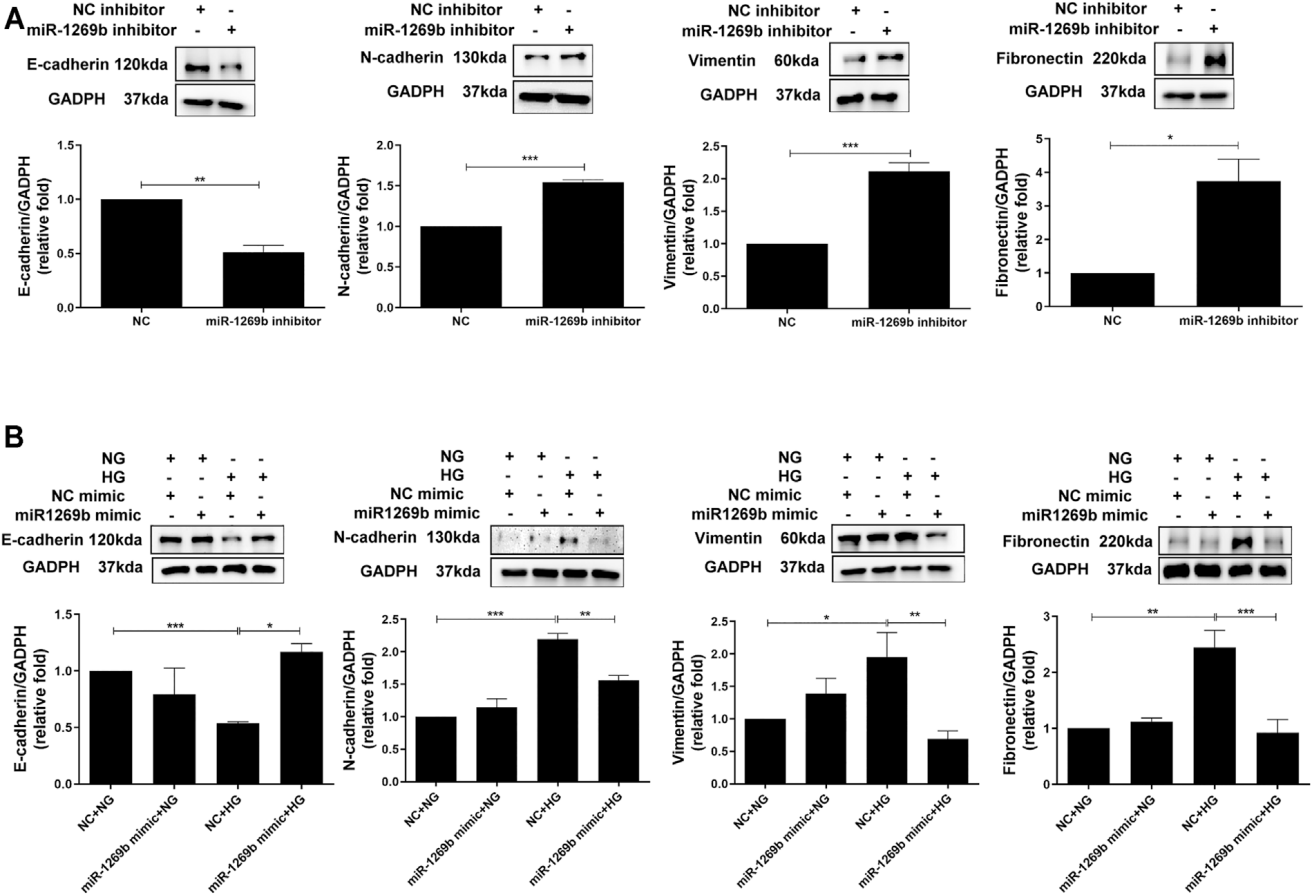
MiR-1269b reduced epithelial–mesenchymal transition (EMT) in proximal tubular epithelial cells (PTECs) of diabetic nephropathy (DN). **(A)** HK-2 cells were transfected with miR-1269b inhibitor (50 nM) or control inhibitor (NC inhibitor, 50 nM) for 24 h and then incubated with normal glucose (NG) or high glucose (HG) for another 48 h. **(B)** HK-2 cells were transfected with either miR-1269b mimic (100 nM) or mimic control (NC mimics, 100 nM). After 24 h post-transfection, cells were cultured under NG condition for 48 h. EMT and extracellular matrix (ECM) expressions were assessed by Western blotting for N-cadherin, vimentin and E-cadherin, and fibronectin. The bar graph represents the mean ± SEM of at least three independent experiments. *p < 0.05, **p < 0.01, and ***p < 0.001 by Student’s *t*-test or ANOVA followed by the post-hoc test with Tukey’s correction.

Since FBLN1 could promote EMT in PTECs, we assessed the biological effect of miR-1269b. After transfection of miR-1269b inhibitor to HK-2 cells, EMT (upregulated N-cadherin and vimentin and downregulated E-cadherin) were induced in HK-2 cells, and fibronectin expression was increased under the NG condition (Figure 4A). In addition, miR-1269b mimic transfection reversed EMT and elevated fibronectin expression in HK-2 cells caused by HG (Figure 4B). These findings suggested that miR-1269b regulated the EMT process and ECM deposition in HK-2 cells.

### Urinary Fibulin-1 Level as a Potential Biomarker of Kidney Injury *In Vivo* Model of Diabetic Nephropathy

**FIGURE 5:**
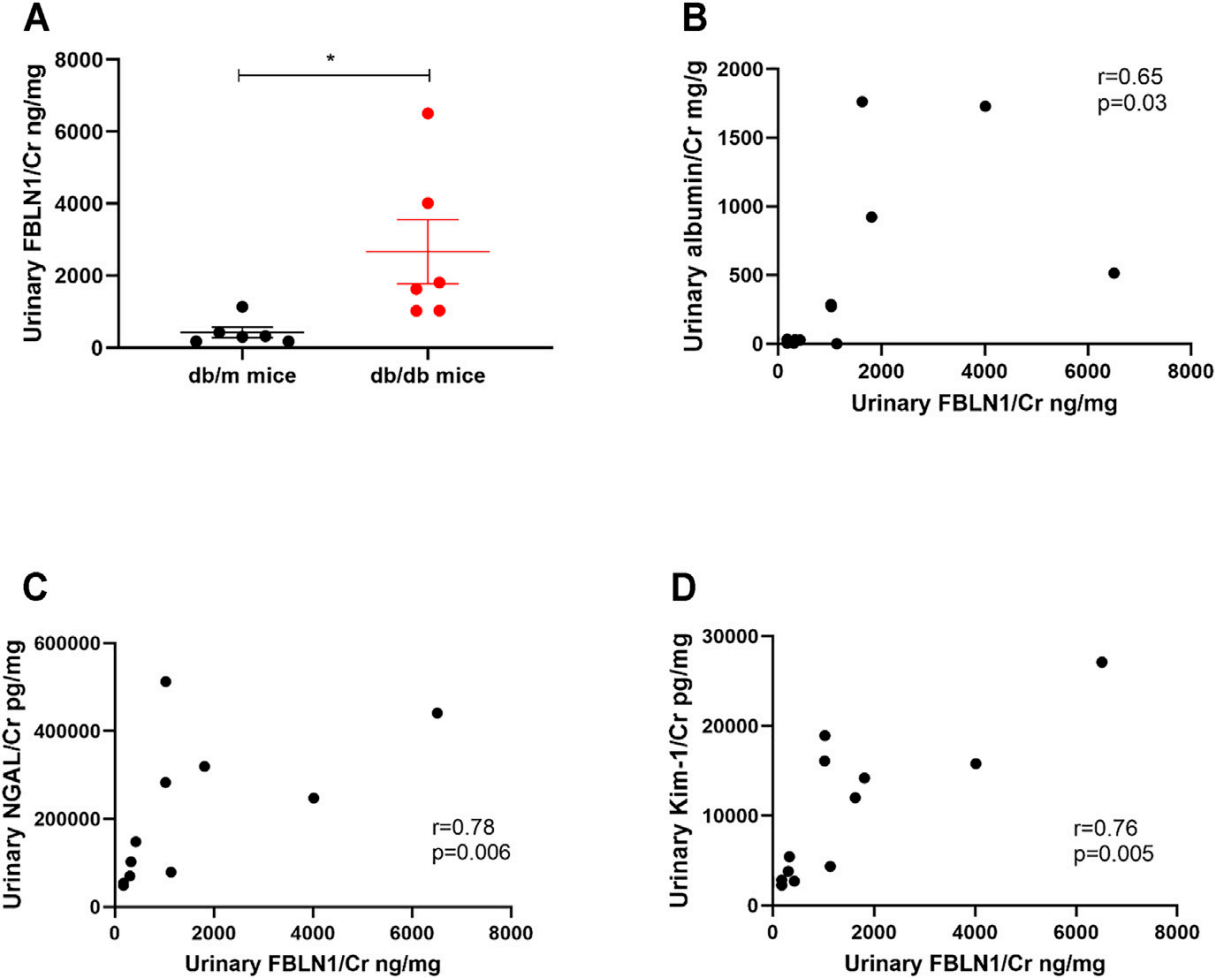
Urinary Fibulin-1 (FBLN1) level as a potential biomarker of kidney injury *in vivo* model of diabetic nephropathy (DN). **(A)** Urinary FBLN1/Cr levels were measured in db/m mice (*N* = 6) and db/db mice (*N* = 6). **(B–D)** The correlations between urinary FBLN1/Cr and albumin–creatinine ratio (ACR), neutrophil gelatinase-associated lipocalin/creatinine (NGAL/Cr), and kidney injury molecule 1/creatinine (KIM-1/Cr) were examined. Urine albumin was measured using an immunoturbidimetric assay. Urine creatinine was determined using the enzymatic method. The levels of FBLN1, NGAL, and KIM-1 in the urine were measured using an ELISA. The bar graph represents the mean ± SEM. *p < 0.05, **p < 0.01, and ***p < 0.001 by Student’s *t*-test. The *p*-value of correlation was analyzed by Spearman’s analysis.

According to the impact of FBLN1 on the EMT process in HK-2 cells under the HG condition, we further assessed the relationship between urinary FBLN1/Cr expression and renal injury *in vivo* model of DN. Urinary FBLN1/Cr levels were higher in db/db mice than db/m mice at the 12th week (Figure 5A). A positive correlation between urinary FBLN1/Cr level and urinary albumin–creatinine ratio (ACR) was found (Figure 5B) in mice. Urinary FBLN1/Cr levels were positively correlated with urinary neutrophil NGAL/Cr and KIM-1/Cr (Figures 5C,D) in mice. Thus, FBLN1 expression in the urine was strongly associated with renal injury markers in the DN animal model.

### Urinary Exosomal MiR-1269b Level and Urinary Fibulin-1 Level as Potential Biomarkers of Kidney Injury in Humans

**FIGURE 6:**
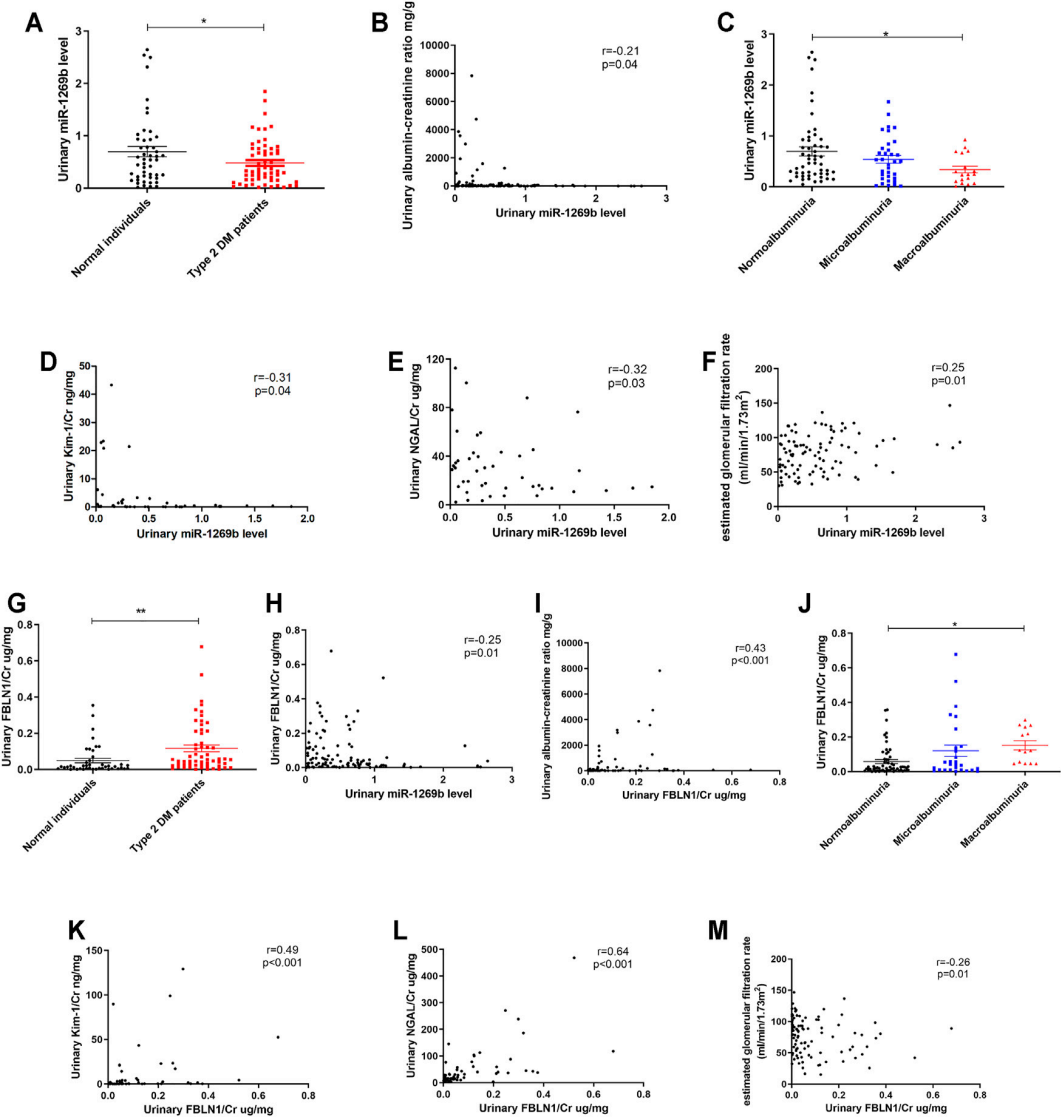
Urinary exosomal miR-1269b level and urinary Fibulin-1 (FBLN1) level as potential biomarkers of kidney injury in humans. **(A)** Urinary exosomal miR-1269b level was measured in normal individuals (*N* = 49) and type 2 diabetes mellitus (DM) patients (*N* = 59). **(B)** The correlation between urinary exosomal miR-1269b level and albumin–creatinine ratio (ACR) was assessed. **(C)** The difference of urinary exosomal miR-1269b levels across the severity of albuminuria in humans. **(D–G)** The association of urinary exosomal miR-1269b levels with kidney injury molecule 1/creatinine (KIM-1/Cr), neutrophil gelatinase-associated lipocalin/creatinine (NGAL/Cr), and estimated glomerular infiltration rate (eGFR) were examined. **(G)** Urinary FBLN1 level was examined in study subjects. **(H,I)** The correlations between urinary FBLN1 levels and urinary exosomal miR-1269b levels and ACR were assessed. **(J)** The difference of urinary FBLN1 level across the severity of albuminuria in humans. **(K–M)** The associations of urinary FBLN1 level with KIM-1/Cr, NGAL/Cr, and eGFR were investigated. Exosomal miR-1269b in the urine of humans was isolated and then assessed by qRT-PCR. Urine albumin was measured using the immunoturbidimetric assay, and urine creatinine was determined using the enzymatic method. The concentrations of FBLN1, NGAL, and KIM-1 in urine were measured using ELISA. Serum creatinine was measured using the compensated Jaffé (kinetic alkaline picrate) method. eGFR was calculated using the equation eGFR = 186 × Serum creatinine^−1.154^ × Age^−0.203^ × 0.742 (if female). The bar graph represents the mean ± SEM. *p < 0.05, **p < 0.01, and ***p < 0.001 by Student’s *t*-test or ANOVA followed by the post-hoc test with Tukey’s correction, and *p*-value of correlation was analyzed by Spearman’s analysis.

Urinary miRNAs have acted as biomarkers for predicting kidney injury (Erdbrugger and Le, 2016). We enrolled 49 normal individuals and 59 patients with T2DM (Table 4) and measured miR-1269b levels in urinary exosomes. T2DM patients had lower urinary exosomal miR-1269b levels than normal individuals (Figure 6A). Urinary ACR was negatively correlated with urinary exosomal miR-1269b (Figure 6B), and individuals with macroalbuminuria had lower urinary exosomal miR-1269b than those with normoalbuminuria (Figure 6C). Urinary exosomal miR-1269b levels were negatively correlated with urinary NGAL/Cr and KIM-1/Cr, as proximal tubular injury markers (Figures 6D,E). Furthermore, we found that low urinary exosomal miR-1269b levels were significantly associated with low eGFR (*r* = 0.25, *p* = 0.001) (Figure 6F).

**TABLE 4:** The clinical characteristics of human participates.

	Normal individuals *N* = 49	Type 2 diabetes *N* = 58	*p*-Value
Age, years	61.8 ± 6.6	63.1 ± 11.6	0.45
Sex (male), %	41.3	65.6	0.01
Fasting blood glucose, mg/dl	94.4 ± 9.7	133.2 ± 35.2	<0.001
Blood urea nitrogen, mg/dl	14.6 ± 3.4	21.4 ± 10.6	<0.001
Serum creatinine, mg/dl	0.7 ± 0.2	1.3 ± 0.6	<0.001
Estimated glomerular filtration rate, ml/min/1.73 m^2^	96.5 ± 19.7	62.0 ± 27.1	<0.001

Note. Data are expressed as number (percentage) for categorical variables and median (25th, 75th percentile) for continuous variables, as appropriate.

In addition, we assessed the relationship between urinary FBLN1/Cr expression, miR-1269b, and renal injury in humans. Urinary FBLN1/Cr levels were conversely correlated with urinary exosomal miR-1269b (Figure 6G). Urinary FBLN1/Cr levels were higher in T2DM patients than in normal individuals (Figure 6H). There was no significant difference in urinary FBLN1/Cr level between males and females (Supplementary Figure S2A–C). A positive correlation between urinary FBLN1/Cr level and urinary ACR was found (Figure 6I), and individuals with macroalbuminuria had higher urinary FBLN1/Cr levels than those with normoalbuminuria (Figure 6J). Urinary FBLN1/Cr levels were positively correlated with urinary NGAL/Cr and KIM-1/Cr (Figures 6K,L). More importantly, high urinary FBLN1/Cr levels were significantly associated with low eGFR (*r* = −0.26, *p* = 0.01) (Figure 6M). In accordance with the results of the *in vitro* and *in vivo* studies, the miR-1269b–FBLN1 axis was shown to participate in the mechanism of HG-induced exosome-mediated kidney injury, suggesting that urinary exosomal miR-1269b and FBLN1 could be used to predict kidney injury in clinical patients with T2DM.

## Discussion

**FIGURE 7:**
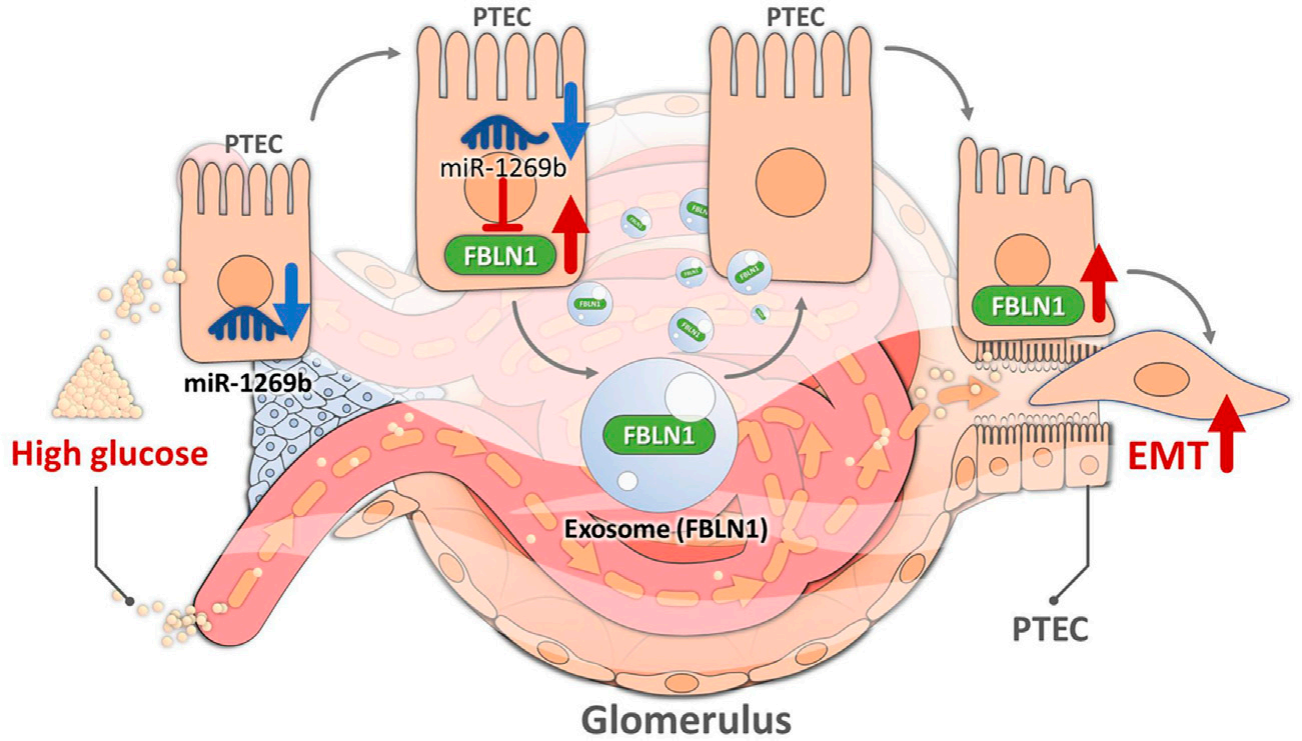
Illustration of the mechanism by which high glucose (HG) induced epithelial–mesenchymal transition (EMT) in proximal tubular epithelial cells (PTECs) through PTEC-derived exosomal Fibulin-1 (FBLN1) in diabetic nephropathy (DN). HG suppressed miR-1269b expression in PTECs, leading to increased expression of FBLN1. FBLN1 further promoted EMT in PTECs through autocrine PTEC-derived exosome delivery.

This cross-disciplinary study demonstrated that exosomal FBLN1 derived from HG-treated HK-2 cells induced pathophysiologic injury in the proximal tubular microenvironment, resulting in DN development. HG-treated HK-2-derived exosomes induced EMT in PTECs through FBLN1, which was modulated via miR-1269b in HK-2 cells. MiR-1269b downregulation led to EMT in PTECs through targeting FBLN1, while miR-1269b upregulation reversed HG-induced EMT in PTECs. Elevated levels of FBLN1 and decreased levels of miR-1269b were found in the urine of mice and patients with T2DM. Furthermore, high levels of urinary FBLN1 and low levels of urinary miR-1269b were correlated with kidney injury in T2DM patients. Taken together, our study provides new insight into understanding the unique cross-talk within the proximal tubules mediated by exosomes and the novel regulation mechanism of miR-1269b–FBLN1 that can be used to explain DN development and be potential biomarkers in clinical T2DM patients (Figure 7).

This study used proteomics analysis to figure out potential exosomal proteins involved in DN mechanisms. We firstly demonstrated that FBLN1 induces EMT in the proximal tubules, further promoting DN progression. Fibulin-1, encoded by FBLN1 gene, belongs to the FBLN1 protein family that is characterized by tandem arrays of epidermal growth factor (EGF)-like domains and a C-terminal FBLN1-type module. FBLN1 is a secreted glycoprotein that stabilizes ECM integrity through interactions with other ECM proteins, such as fibronectin (Segade, 2010). FBLN1 also regulates cell adhesion and mobility, and matrix remodeling (Argraves et al., 1990; Tran et al., 1997). Previous studies indicated that elevated expression of FBLN1 is associated with cancer progression, including hepatocellular carcinoma, cholangiocarcinoma, and renal cell carcinoma (Xiao et al., 2013; Jie et al., 2019; Gong et al., 2020). FBLN1 has been involved in airway remodeling in chronic asthma and pulmonary fibrosis (Liu et al., 2016). In addition, Scholze et al. found that elevated circulating FBLN1 levels were positively associated with cardiovascular risks in patients with CKD and T2DM (Scholze et al., 2013). However, the evidence of FBLN1 in the pathogenesis of DN is limited. Our study proved that HG elevated proximal tubule-derived exosomal FBLN1 expression, which in turn induced EMT and ECM accumulation in the proximal tubules by an autocrine model. Silencing FBLN1 reversed EMT and ECM accumulation in the proximal tubules. Furthermore, high urinary FBLN1 levels were closely correlated with the severity of renal injury in T2DM patients. Thus, FBLN1 not only participates in the pathogenesis of DN via exosome transduction but also could be a potential biomarker of kidney injury in DN.

Accumulating evidences indicated that exosomes are not only emerging as significant mediators in the pathogenesis of DN but also showing their potential in prognostic and therapeutic applications in DN (Alicic et al., 2017). Following their secretion, exosomes can act on their cells of origin in an autocrine manner, on cells in their vicinity in a paracrine manner, and on cells in distant organs. To exert their effect, exosomes may bind to the surface of their target cells and signal through cell surface receptors. Alternatively, they may enter target cells via endocytosis or by direct fusion with the plasma membrane of the target cells and thereby release their cargo (Zhang et al., 2019). FBLN1 has been shown to interact with integrins or ECM proteins, such as proteoglycan (aggrecan or versican). FBLN1 could bind to the cytoplasmic domain of integrin β1, which in turn activates focal adhesion kinase (FAK) and Yes-associated proteins (YAP), resulting in EMT in various tissues (Twal et al., 2015; Liu et al., 2017; Zhou et al., 2020). Our results showed that HK-2 cells could uptake the exosome released from PTECs under HG stimulation, suggesting that exosomal FBLN1 may possibly bind to the cytosol integrin β1 and subsequently trigger FAK cascade to promote EMT in recipient cells. Our current findings demonstrated that exosomal FBLN1 derived from PTECs promoted EMT in PTs, meaning that FBLN1 might mediate the cross-talk between PTECs through autocrine exosome transmission. Further studies are necessary to examine the pivotal role of integrin and FAK in mediating exosomal FELN1 transmission of DN.

On the other hand, urinary exosomes and their biomaterials are released by all cell types along with kidney structures and carry valuable information for the stage-specific prognosis of kidney diseases (Erdbrugger and Le, 2016). Urinary exosomes could be noninvasive and sensitive biomarkers to diagnose and predict kidney diseases (Miranda et al., 2010; Gudehithlu et al., 2015). In the present study, we found that urinary exosomal miR-1269b was correlated with the severity of kidney injury in T2DM patients. In addition, miR-1269b not only regulated FELN1 expression in PTECs of DN but also reversed the FBLN1-induced EMT process and ECM accumulation in DN. These findings prove the impact of miR-1269b on pathophysiologic mechanisms of DN and suggest the potential use of exosomal miR-1269b in clinical biomarkers of DN.

In conclusion, exosomal FBLN1 derived from PTECs under HG induced EMT and ECM accumulation in PTECs through miR-1269b modulation. The cross-talk among PTECs by exosomes contributed to DN progression. MiR-1269b–FBLN1 epigenetic regulatory network may be a potential therapeutic strategy for DN.

## Data Availability

The datasets presented in this study can be found in online repositories. The names of the repository/repositories and accession number(s) can be found in the GEO database GSE185586.

## Abbreviations

ACR, albumin-creatinine ratio; CKD, chronic kidney disease; CM, conditional medium; DAVID, Database for Annotation, Visualization, and Integrated Discovery; DM, diabetes mellitus; DN, diabetic nephropathy; eGFR, estimated glomerular filtration rate; EMT, epithelial-mesenchymal transition; ESRD, end-stage renal disease; EV, extracellular vesicular; EVs, extracellular vesicles; FBLN1, fibulin-1; FN1, fibronectin-1; HEK, human embryonic kidney; HG, high glucose; HK-2, human kidney-2; IHC, immunohistochemistry stain; IPA, Ingenuity Pathway Analysis; KIM-1, kidney injury molecule 1; miRNA, microRNA; NG, normal glucose; NGAL, neutrophil gelatinase-associated lipocalin; NGS, next-generation sequencing; PBS, phosphate-buffered saline; PTEC, proximal tubular epithelial cell; RIPA, radio-immunoprecipitation assay; RT-Q-PCR, reverse transcriptase quantitative real-time PCR; TBS, Tris-buffered saline; 3′UTR: 3′-untranslated region
