# Supplementary material for: Autocrine Exosomal Fibulin-1 as a Target of MiR-1269b Induces Epithelial–Mesenchymal Transition in Proximal Tubule in Diabetic Nephropathy
Source: Front Cell Dev Biol. 2021 Dec 17;9:789716. doi: 10.3389/fcell.2021.789716 (PMC8718747; doi:10.3389/fcell.2021.789716)
Supplement: Supplementary file 1 [file DataSheet1.PDF]

Table S1. Target sequence of materials utilized in the study.

description	Mature microRNA sequence
Smart Pool ON-TARGETplus Fibulin-1 siRNA	Target Sequence:<br>GCGAAUGCAAGACGGGUUA<br>GAGGGAACGCGCUGUGUUG<br>AAUGAGUGUUUGAGUAUCA<br>AUAAGAUGAUUGAGGUUGA
Smart Pool ON-TARGETplus Non-targeting siRNA	Target Sequence:<br>UGGUUUACAUGUCGACUAA<br>UGGUUUACAUGUUGUGUGA<br>UGGUUUACAUGUUUUCUGA<br>UGGUUUACAUGUUUUCCUA
miRIDIAN microRNA Human has-miR-1269b-Mimic	Mature microRNA sequence<br>CUGGACUGAGCCAUGCUACUGG
miRIDIAN microRNA Human has-miR-1269b-Hairpin Inhibitor	Mature microRNA sequence<br>CUGGACUGAGCCAUGCUACUGG
hsa-miR-1269b	5'd CTGGACTGAGCCATGCTACTGG 3'

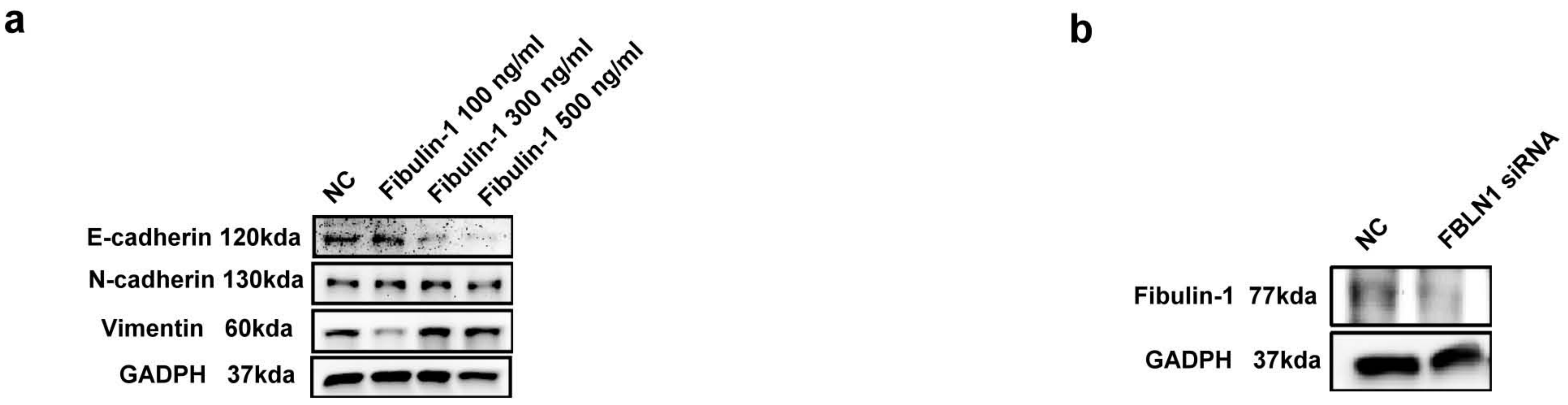
a
NC
Fibulin-1 100 ng/ml
Fibulin-1 300 ng/ml
Fibulin-1 500 ng/ml
E-cadherin 120kda
N-cadherin 130kda
Vimentin 60kda
GADPH 37kda
b
NC
FBLN1 siRNA
Fibulin-1 77kda
GADPH 37kda

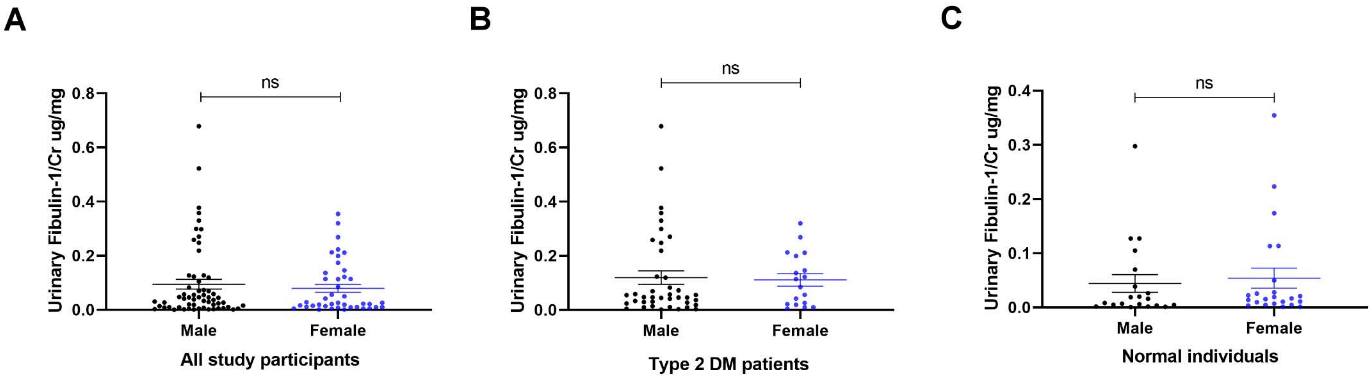

Supplementary Figure 2
